# The Structure of Mutations and the Evolution of Cooperation

**DOI:** 10.1371/journal.pone.0035287

**Published:** 2012-04-26

**Authors:** Julián García, Arne Traulsen

**Affiliations:** Research Group for Evolutionary Theory, Max-Planck-Institute for Evolutionary Biology, Plön, Germany; Hungarian Academy of Sciences, Hungary

## Abstract

Evolutionary game dynamics in finite populations assumes that all mutations are equally likely, i.e., if there are 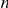 strategies a single mutation can result in any strategy with probability 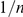. However, in biological systems it seems natural that not all mutations can arise from a given state. Certain mutations may be far away, or even be unreachable given the current composition of an evolving population. These distances between strategies (or genotypes) define a topology of mutations that so far has been neglected in evolutionary game theory. In this paper we re-evaluate classic results in the evolution of cooperation departing from the assumption of uniform mutations. We examine two cases: the evolution of reciprocal strategies in a repeated prisoner's dilemma, and the evolution of altruistic punishment in a public goods game. In both cases, alternative but reasonable mutation kernels shift known results in the direction of less cooperation. We therefore show that assuming uniform mutations has a substantial impact on the fate of an evolving population. Our results call for a reassessment of the “model-less” approach to mutations in evolutionary dynamics.

## Introduction

Evolutionary game dynamics can be used to study the evolution of phenotypes. It usually considers the fate of a population of strategies playing a game, subject to selection and mutation. In this framework one of the most studied formalisms is the Moran process, it allows for studying the interplay between selection and mutation under demographic noise. The Moran process considers a finite population of constant size. At every time step one strategy is chosen for reproduction in proportion to its performance in the current population. A copy of this strategy is added to the population after removing a random strategy. With a small probability, the strategy that is copied changes its type to any of the other available strategies. This process results in an ergodic Markov chain. The effect of selection and mutation can be assessed by inspecting the average composition of the population in the long run.

The Moran process is often studied in the limit of small mutation probability [1], [2]. A number of key results have been derived in such a setting, particularly in the literature that concerns the evolution of cooperation [3]–[10]. In these studies, mutations are usually assumed to be uniform, such that any strategy can mutate to any other with the same probability [11]. Non-uniform mutations arise when these probabilities vary, and not all states are reachable from a given population, or certain states are easier to reach than others. Considering such asymmetries can dramatically change the outcome of evolution [12]. In this paper, we study how and illustrate it with a few examples. We find that even if mutations are rare, the structure of how mutations arise from the different types matters.

Evolutionary processes have been traditionally given two possible interpretations. In cultural evolution, the process of selection is taken to represent a situation in which successful strategies spread by imitation. Here, mutations are generally interpreted as mistakes in the process of imitating others, or intended exploration undertaken by individuals [13]. The idea of non-uniform mutations means in this interpretation, that individuals may be more prone to explore strategies that are less costly to implement, strategies that imply less risky outcomes, or strategies that are similar to their previous strategies. Another interpretation is genetic. Here it would seem natural that not all mutations can arise from a given state. Certain mutations may be far away, as a consequence of the complexity of mutation processes and the (mostly unknown) intricacies of how genes code for different phenotypes. For our study, we do not need to specify in detail whether cultural or phenotypic evolution is considered.

Some previous studies have already considered non-uniform mutation rates. The idea of local mutations is central in adaptive dynamics, but here the literature is strictly concerned with infinite populations and continuous strategies in metric spaces [14], [15]. Also for evolutionary games in infinite and finite populations with discrete strategies, general results have been obtained [16]–[19]. Fudenberg et al. [20] provide a general analysis for 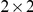 games in finite populations under arbitrarily small mutations and non-weak selection. Imhof and Nowak explore the idea of local mutations in the continuous strategy space of reactive strategies for direct reciprocity [21]. Bergin and Lipman [12] argue that mutations should be specifically modeled, any refinement effect coming from the uniqueness of a stationary distribution is due to the specific assumptions made about mutations. Binmore and Samuelson [22] analyze the effect of different mutation rates in a non-generic game called the resource game. Their analysis is focused on deterministic dynamics in infinite populations where the rest points are clustered. Also in a deterministic setting, Willensdorfer and Nowak [23] inspect the effect of (different) mutation rates in average population fitness. None of these studies goes further into specifying how the different rates of mutation could vary. Here we introduce a direct comparison between different mutation kernels in the same game.

We analyze mutation structures in two examples dealing with the evolution of cooperation. We restrict ourselves to mutation structures that are non-frequency dependent and do not vary with time. Our examples resemble the concept of a “protein space”, as envisioned by Maynard Smith [24], [25]. Here, phenotypes (strategies) live in a hypercube and each mutation step represents a local change in an underlying chain of amino-acids, represented by strings made up of a finite number of bases. We show that known results are already drastically called into question when considering mutation structures beyond the standard case of uniform mutation rates.

## Results

### Mutations matter, even when they are rare

Consider the simple case of competition between two strategies 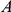 and 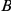. To study non-uniform mutations, we introduce two mutation rates: 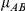 is the probability that an 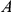 type mutates into a 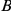 type, and 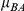 is the probability that a 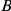 type turns into an 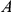 type.

The evolutionary dynamics is considerably simplified for small mutation rates. If mutations are small enough [2], mutants arise whenever the population has fixated on a strategy, and the dynamics can be completely characterized by studying a Markov chain between monomorphic states [3], [11]. This transition matrix is
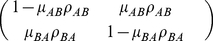
(1)where 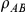 is the fixation probability of a single mutant 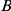 in a population of 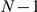 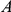
's, and 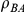 is the fixation probability of single mutant 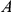 in a populations of 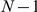 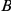
's. The long-term dynamics of the system is described by the stationary distribution
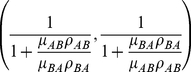
(2)This implies that 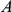 is more abundant than 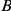 in the long run when
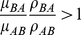
(3)Let us further specify what 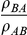 looks like. Consider a game with payoff matrix
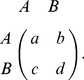
(4)The expected payoffs when there are 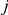 A players in the population are
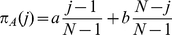
(5)

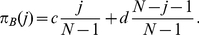
(6)Following [26], we map payoff to fitness using an exponential function
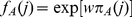
(7)

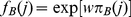
(8)where 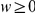 is the intensity of selection. For a standard Moran process without mutations the fixation ratio is
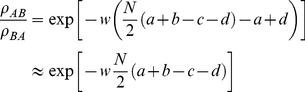
(9)where the approximation is valid for large 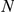 [27]. With equation (3), 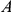 is more abundant than 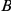 in the long run, when
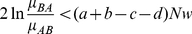
(10)


For 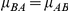 we obtain the usual risk-dominance condition [28]. But equation 10 implies that it is always possible to choose a ratio of mutation probabilities, such that any disadvantage in the game can be reversed by asymmetries in mutations. These asymmetries only have an effect when selection is not infinitely strong, 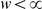. Note that 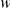 and 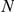 enter linearly, but 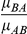 features in a logarithmic fashion.

These results hold for any finite intensity of selection and small (positive) mutations. Even for small mutation rates, the specific model of how mutations arise can dramatically change the fate of an evolving population, as shown previously in [20].

Usually, the mutation rate is one single parameter. In larger systems, studying non-uniform mutation rates requires us to specify how likely it is that any given strategy 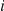 will mutate into any other possible strategy 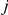. Hence, mutation rates can be defined by a stochastic matrix 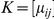 such that position 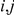 specifies what the probability is to mutate from strategy 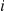 into strategy 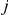. We call such matrix a mutation kernel. Each row of this matrix is normalized such that 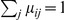. Accordingly, completely specifying the mutation structure requires 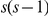 numbers for a strategy set of size 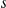.

Let us now look at how such kernels may be specified for particular examples, and how known results do change when departing from uniform mutations.

### Direct reciprocity: the repeated prisoner's dilemma

We start by studying the evolution of direct reciprocity [29]. In the one-shot prisoner's dilemma defection is the only stable outcome of the game. Cooperation can be stable, however, if the game is repeated and the possibility of retaliation exists. This mechanism is usually referred to as direct reciprocity. As opposed to the dichotomous choice in the one shot version, repetition opens many possibilities. We focus on deterministic strategies with a finite – albeit uncertain – horizon. A strategy for a repeated game specifies which action to play, given the history of the game so far. In a repeated game, for any two strategies 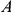 and 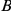 the payoff of 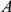 when it faces 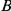 will be computed as
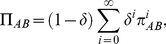
(11)where 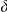 is the continuation probability, and 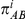 is the pay-off in the 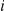-th round of the game. For convenience we have normalized the pay-off of the repeated game multiplying by 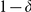.

The one shot game we are interested in is a prisoner's dilemma with the pay-off matrix 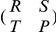, with 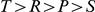 and 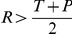. The literature on the repeated prisoner's dilemma is extensive [8], [21], [30]–[34]. For instance, Imhof et al. [8], study a subset of 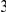 strategies: always cooperate (*ALLC*), tit-for-tat (*TFT*) and always defect (*ALLD*). Strategies *ALLC* and *ALLD* stand for unconditional cooperation and defection respectively; *TFT* cooperates in the first move, and then copies what the opponent did in the last round. Computing the payoff for this set of strategies, according to equation (11), yields matrix
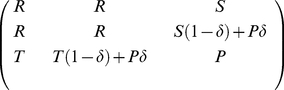
(12)


A finite population will spend most of the time in the cooperative strategy *TFT*, provided a sufficiently large continuation probability [8]. Even though studying this subset of strategies is insightful, it can be argued that it is a biased subset: in neutrality, cooperative behaviour is overrepresented because 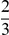
's of the time are spent in strategies that are completely cooperative. Moreover, within this subset, it is difficult to come up with mutation kernels that differ from the uniform one without being completely arbitrary.

The complete set of strategies for the repeated prisoner's dilemma is infinite. Therefore, studying a particular dynamics implies some restriction in the strategy set. In this section we study the 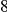 strategies described in Table 1. This is the deterministic subset of the strategies considered in [35]; it contains all possible deterministic strategies that consider the opponent's last move. Therefore, a strategy is completely determined by three pieces of information. The first item determines the action to take on the first move. The second item is what to do if the opponent cooperated, and the third one dictates what to do upon the other defecting. As we will see, this way of conceiving the strategies will further provide a straightforward alternative mutation structure.

**Table 1:** Strategy set in the repeated prisoner's dilemma with one round memory.

	Strategy	Behavior	Binary code
0	*ALLC*	Always cooperate	000
1	*TFT*	Tit for tat	001
2	*TFT* 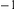	Cooperate on the first move	010
		then reverse the opponent's last move	
3	*NALLD*	Cooperate once and then always defect	011
4	*SALLC*	Defect once and then always cooperate	100
5	*STFT*	Defect once and then copy the opponent's last move	101
6	*STFT* 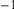	Defect once and then reverse the opponent's last move	110
7	*ALLD*	Always defect	111

For the uniform mutation kernel, it is convenient to number the strategies with integers, for the bitwise kernel binary numbers are more convenient.

The derivation of the pay-off matrix for all the strategies in Table 1 is given in Section A of the Supporting Information Text S1. We compute the abundance in the long run, as described in the Methods section. That is, we study a Moran process with exponential fitness mapping in the limit of rare mutations. The validity of the theoretical prediction here depends on an appropriate choice of population size (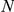) and continuation probability (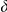), that guarantees that mutations are sufficiently slower than fixation events. In order to comply with such requirement we restrict ourselves to large values of 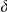, see Section B of the Supporting Information Text S1.

#### Uniform mutations

**Figure 1:**
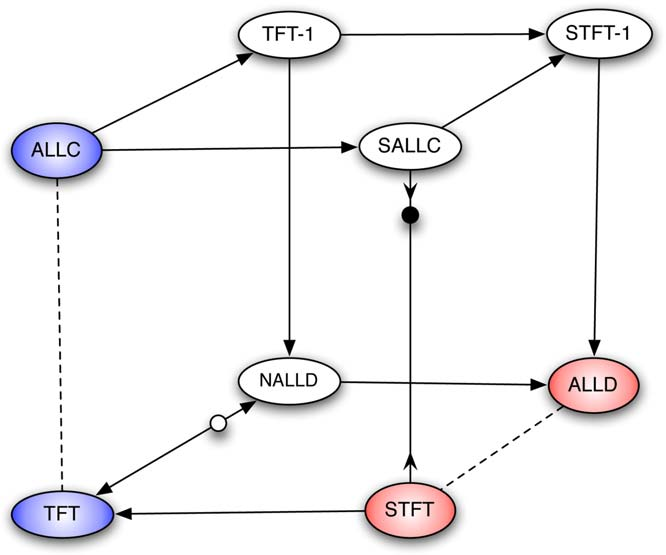
Repeated prisoner's dilemma: Average abundance in stationarity. Panel A shows uniform mutations, and Panel B shows the results for the bitwise kernel. Continuous lines represent the theoretical approximation. Dots represent simulation results averaged over 500 repetitions of 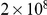 generations each, and a mutation probability 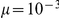. Plus signs represent a larger mutation probability, 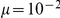. In this case of larger 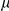, which is harder to address analytically, the mutation kernel also affects the average abundance. Values for the game are 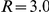, 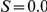, 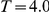, 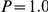. The continuation probability is 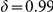, and population size is 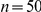.

For the uniform mutation structure, a mutation occurs with probability 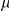, and all other strategies have the same chance to be the result of one mutation step. The strategy chosen for reproduction does not undergo mutation with probability 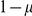. The mutation kernel is thus an 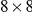 matrix with 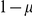
's in the diagonal elements, and 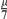 elsewhere. All strategies are reachable from each other via mutations. Panel A in Figure 1 shows the results with the standard assumption for population size 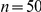. Strategy *TFT* is by far the winning strategy in a large region of the parameter space. This is consistent with the findings in [8]. Unconditional defection is the most popular strategy only for very strong selection.

#### Bitwise mutation

**Figure 2:**
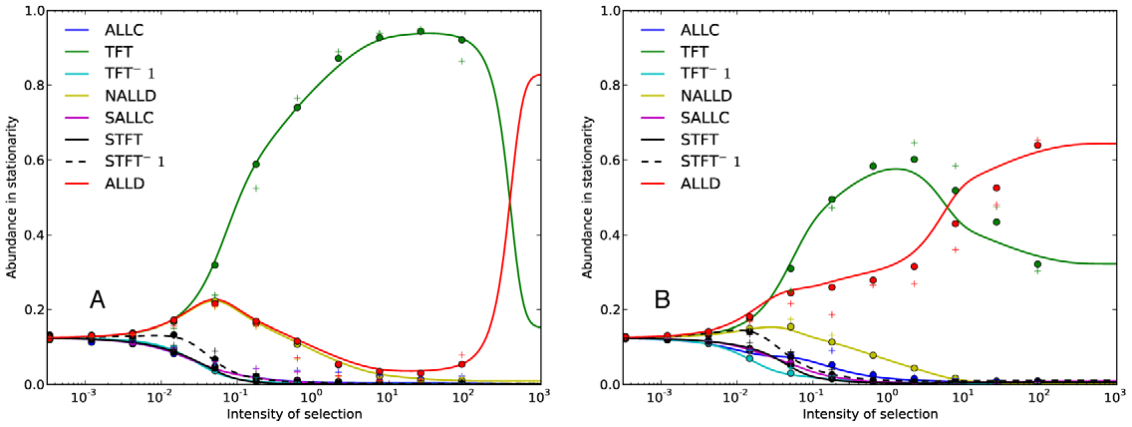
Repeated prisoner's dilemma: structure of selection and mutation for bitwise mutation. Arrows indicate the direction of selection, and dashed lines indicate neutral paths. Blue strategies are completely cooperative and red strategies are completely uncooperative when paired with themselves. The kernel structure shuts down paths that would normally be available with the standard assumption that all mutation paths are possible.

We now assume that strategies are represented by the binary code, as described in Table 1. The digit 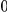 stands for cooperation, and the digit 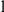 stands for defection. The first digit codes for the initial action, the second digit determines what to do upon cooperation and the third digit determines what to do upon defection. This binary representation is common in disciplines like evolutionary computation [36]. Possible mutations are those between any two strategies that differ in one bit. Thus, the associated mutation kernel is given by
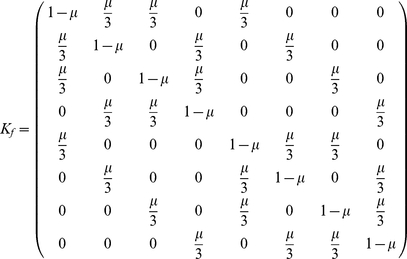
(13)Any strategy in this kernel has only three neighbors, thus the mutation matrix is sparse. The structure of selection and mutation is depicted in Figure 2.

**Figure 3:**
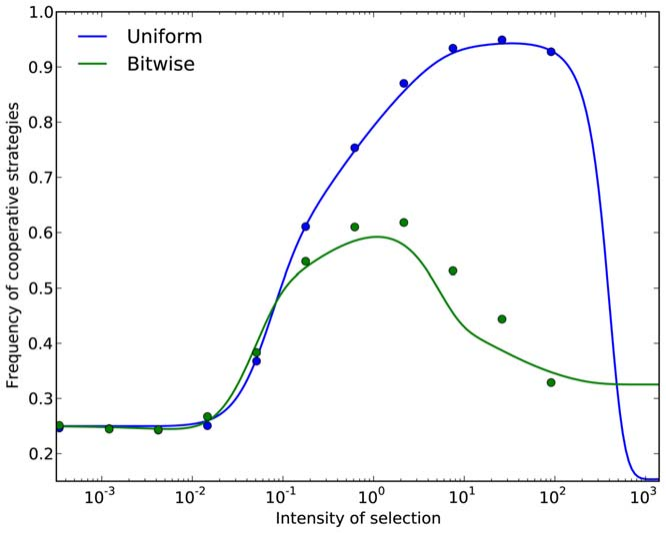
Repeated prisoner's dilemma: Fraction of time spent on fully cooperative states in the stationary distribution. 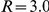
, 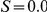, 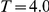, 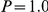. The continuation probability is 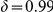, population size is 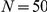, and mutation probability is 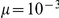. Continuous lines are theoretical approximations for small mutation rates and dots represent simulation results.

The results are shown in Panel B of Figure 1. Compared to the case of uniform mutations, the region of the parameter space where *TFT* is the most popular strategy has been sharply reduced. In particular, *ALLD* is able to beat *TFT* at a much lower intensity of selection. In this case, we see that a reasonable mutation kernel substantially reduces cooperation in the long run. This is illustrated in Figure 3. Our results are consistent with the findings of [21], where local mutation reduces the abundance of cooperative strategies. The main difference with that study is that we study a finite strategy set in a game that is not indefinitely repeated, and that our notion of locality naturally stems from the binary representation of strategies.

The results of the bitwise kernel are of course invariant to changing the meaning of each position (e.g., the last bit, instead of the first, determines what to do in the first round), or changing the meaning of each bit (e.g., cooperation is coded by 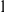 instead of 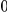). Neighborhoods are preserved under any scheme that codes the strategies in a binary fashion.

Note that arbitrary kernels can produce results that differ in more radical ways from the standard result with uniform mutations. For instance, changing the labels by swapping strategies *STFT* 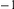 and *NALLD* in Figure 2, will cause *SALLC* to be the most popular strategy under strong selection. With such a choice, *SALLC* turns out to be the only strategy that dominates all its neighbors. The appealing feature of the bitwise kernel is that it naturally stems from considering a binary representation of the strategies. As depicted in Figure 3, the idea that direct reciprocity leads to high levels of cooperation rests on one particular choice of mutation kernel.

### Optional public good games with punishment

We now turn to cooperation without repetition. The evolution of strategies in the optional public goods game has been studied extensively since proposed by Fowler [37]. The model considers four types: cooperators, who invest a given endowment in a joint enterprise; defectors, who do not invest in the public good but benefit from it; punishers, who cooperate and in addition punish those who do not cooperate; and loners, who get a fixed payoff abstaining from the game. The model has been refined in a series of papers [3], [7], [38]–[40]. The main result for finite populations is that the system spends a considerable amount of time in cooperative states. The threat of punishment opens the door for cooperation, which is stabilized by the option of abstaining. Even though loners do not have a large share in the stationary distribution, their presence is essential to maintaining cooperation [3].

Here, we will follow the version of the game presented in [7]. There is a well-mixed finite population of size 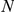. At every time step, individuals get into groups of size 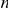. Within these groups they have the option to play a public goods game. Those who participate can decide wether to invest or not in a joint enterprise, at a cost 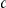. The total sum of the pot is multiplied by a factor of 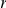, and divided equally between those who took part in the game. Loners, i.e., individuals that abstain, get a fixed payoff 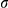. After this interaction, each contributor can impose a fine 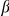 upon each defector, assuming a cost 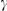 for each fine. The expected payoff follows [7]. As in previous sections, we will inspect abundance in stationarity for a Moran process with exponential mapping in the limit of rare mutations.

#### Uniform mutations

**Figure 4:**
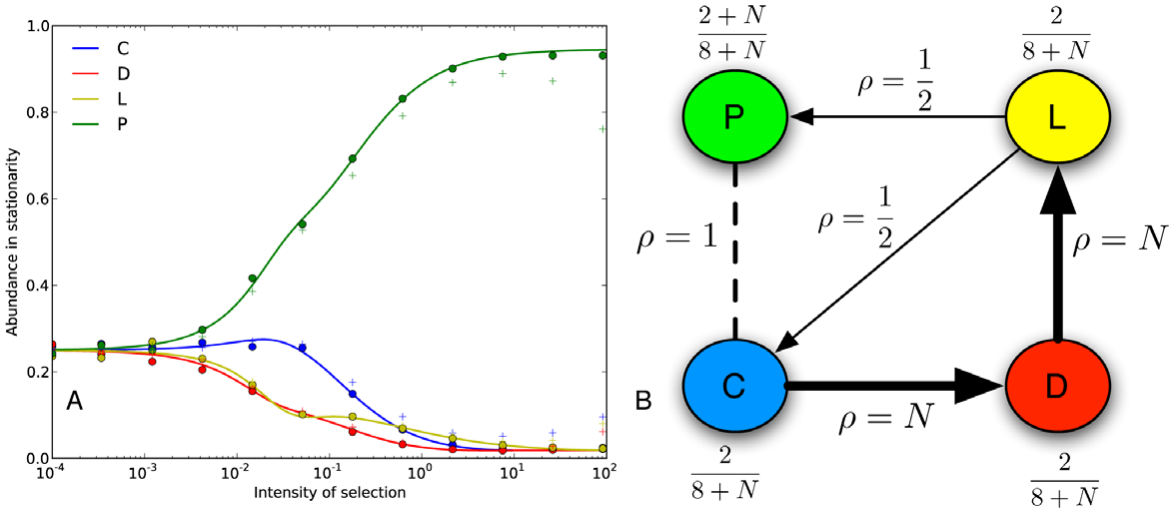
Optional public goods game with uniform mutations. Panel A shows abundance in stationarity as a function of the intensity of selection. Continuous lines represent the theoretical approximation. Dots represent simulation results averaged over 500 repetitions of 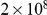 generations each, and a mutation probability 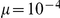. Plus signs represent a larger mutation probability, 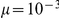. 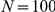, 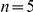, 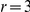, 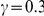, 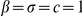. Panel B shows transitions (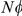) between monomorphic states and abundance as a function of population size in the limit of strong selection (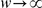).

For uniform mutations the systems spends most of the time in a population completely made up of cooperators that punish defectors. This can be seen in Figure 4 (panel A), where we show the abundance in stationarity, as a function of the intensity of selection. Clearly, selection leads to the prevalence of altruistic punishers.

Analytical results greatly simplify in the limit of strong selection (i.e., 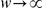), where all the fixation probabilities reduce to 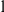 , 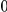, 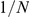 or 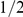 [7]. In this limit the stationary distribution is given by
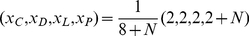
(14)


Figure 4 (panel B) shows the stationary distribution, as well as the relative speed of transitions between homogeneous states in the limit of strong selection. Punishers are vulnerable to invasion via neutral drift by cooperators, which in turn are susceptible to invasion of defectors. But loners offer a way out of defection and back into cooperators or punishers. Thus, via freedom to coercion [3]. On average, in the long run, altruistic punishers are most abundant, and cooperation is sustained.

#### Slower transitions towards sociality

We can depart from the standard assumption of uniform mutations, for instance, assuming that mutations from loners towards the other strategies are rarer by a factor 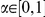. This mutation kernel is given by matrix 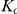.
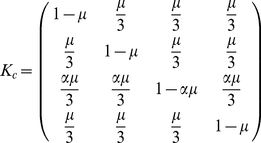
(15)


We can provide a straightforward interpretation for this mutational structure, biologically as well as from a cultural perspective. Biologically, mutations towards strategies that do actually play the game (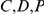) could be rarer, since the demands of social living may require a number of specific mutations to accumulate before individuals can cope with such demands. On the other hand, in cultural terms, the factor 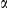 could be thought of as a measure of risk aversion. Given that playing the game is risky, agents are more hesitant to jump into strategies that carry such risk. A low 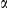 value would mean that loners are less prone to jump into the game.

In the limit of strong selection the calculations are again greatly simplified. The stationary distribution is given by:
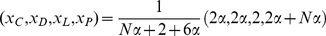
(16)


Thus, in the limit of strong selection, playing the game is more popular than abstaining whenever 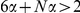.

**Figure 5:**
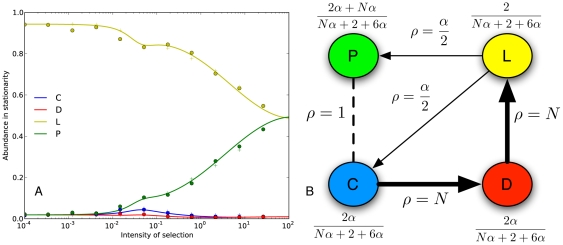
Optional public goods game with non-uniform mutations. Panel A shows abundance in stationarity as a function of the intensity of selection (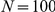, 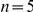, 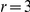, 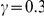, 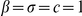) using kernel 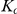 with 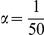. Continuous lines represent the theoretical approximation. Dots and plus signs represent simulation results. Panel B shows transitions (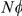) between monomorphic states and abundance as a function of population size, using kernel 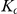, in the limit of strong selection (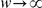).

Figure 5 shows in panel A, the abundance in stationarity as a function of the intensity of selection. The value of 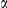 is 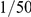. In panel B, we show the transitions and the stationary distribution using this kernel in the limit of 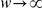. For 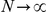, social individuals will be more popular for any 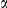. But population size introduces a limit in which risk averse individuals refrain from playing the game. The reason is that transitions out of the asocial state can be considerably slower.

Accordingly, we show that in finite populations some risk aversion may deter cooperation, as most individuals prefer not to play the game. This is radically different from what happens in the case of uniform mutations, where strong selection always leads to total predominance of altruistic punishers. Other mutation structures are of course possible, but once again, it is difficult not to be completely arbitrary. In the next section we inspect a bitwise mutation kernel for this game.

#### Bitwise-like mutations in a larger strategy set

A larger strategy set for the optional public good games with punishment has been recently studied by Rand and Nowak [6]. In this study, individuals can contribute to the public good game 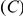, play avoiding contribution 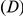 or abstain from playing 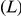. In addition, they can decide wether to punish or not each of the other types. A strategy is then a 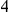-tuple 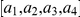, where 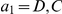 or 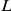, and 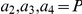 or 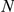. Element 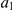 codes for contribution or abstention, 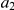 determines whether to punish or not cooperators, 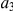 determines whether to punish or not defectors, and 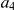 determines whether to punish or not loners. The strategy set composed of 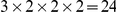 strategies. This strategy set provides the possibility of antisocial punishment, that is, non-cooperators that punish cooperators.

The game has the same parameters and structure as the game considered above; the only difference comes in the specification of payoffs for each one of the 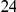 strategies. The formulas are given in detail in the appendix of [6]. We perform the same type of analysis, that is, we inspect the stationary distribution that comes from a Moran process with exponential mapping, in the limit of rare mutations.

We compare the uniform mutation structure, where all strategies can be reached from each other with the same weight, with a bitwise-like kernel that has the following structure. Each strategies is a chain of four positions. The first position has base 3: D stands for defection, C stands for cooperation, and L stands for loner. The second, third and fourth positions are binary. P in the second position means punish cooperators, whereas N means do not punish cooperators. The third position takes care of punishing defectors, and the fourth position codes for punishing loners. In the bitwise-like kernel, mutations can only take you to a strategy that differs in one position. This means that each strategy has 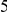 neighbors as opposed to 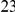 in the uniform case.

For example, defectors who do not punish (D-NNN) can only mutate into defectors that punish loners (D-NNP), defectors that punish other defectors (D-NPN), defectors that punish cooperators (D-PNN), cooperators that do not punish (C-NNN) and loners that do not punish (L-NNN). Each one of these events happens with probability 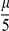, and there is no mutation with probability 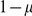.

**Figure 6:**
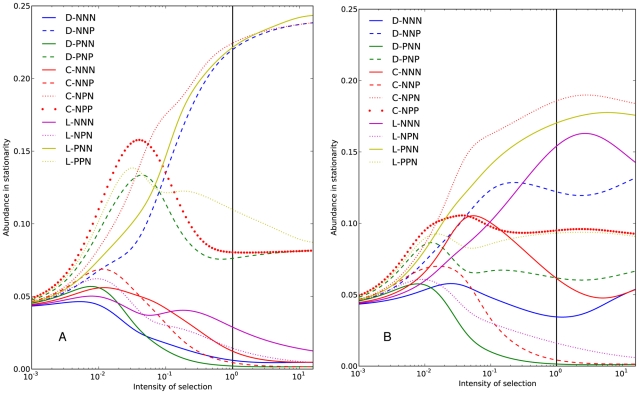
Optional public goods game with bitwise-like mutations. Abundance in stationarity as a function of the intensity of selection (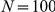, 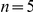, 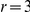, 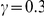, 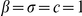). Panel A shows results for uniform mutation structure. Panel B shows results for bitwise-like mutations. The reference intensity of selection (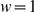) is marked with a vertical black line.

Figure 6 shows the results of the analysis for both kernels. For the sake of clarity we exclude self-punishing strategies, whose frequency in stationarity is very low. Following [6], let us focus on what happens for intensity of selection equal to 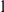 (solid vertical black line). For uniform mutations it is clear that no strategy is overwhelmingly prevalent. All strategies are below 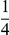. The three most popular strategies are: C-NPN, L-PNN, and D-NNP. The system spends more than 60% of the time on these three strategies. The most popular strategy corresponds to altruistic punishers, followed by loners that punish defectors, and defectors that punish loners. It is striking that antisocial punishment is associated mostly to asocial individuals who abstain from playing the game.

Introducing the bitwise-like mutation structure we also find that no strategy is overwhelmingly prevalent. In particular, the stationary distribution has more variation and other strategies become abundant. The most popular strategies are now altruistic punishers, loners that punish cooperators, and individuals that refrain from taking any action whatsoever. It is noteworthy that introducing this kernel considerably favors the autarkic option of individuals that abstain from the game, and forgo any punishment.

## Discussion

We have formalized a Moran process with non-uniform mutations. We show that mutation structure plays an important role, even if mutations are assumed to be small. In three examples we have come up with specific reasonable kernels that overturn known results. Our mutation kernels are akin to Maynard-Smiths's concept of protein spaces, where phenotypes are connected by unit mutational steps [24], [25].

We first study the evolution of direct reciprocity in a set of 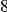 strategies. Representing strategies as strings of bits, we introduce a new mutation structure that reduces significantly the amount of cooperation to be expected in the long run.

Next we turn to a model of cooperation without repetition. We study the evolution of altruistic punishment in optional public good games. Assuming a reasonable kernel with a clear biological and behavioural interpretation leads to the possibility of abstention being more successful than playing the game. A specific condition is specifically worked out for the case of strong selection. Finally, we study optional public good games with punishment in a much larger strategy space. The structure of the space also lends itself to an interpretation that makes it easy to come up with a reasonable mutation kernel. This kernel changes the results in a significant manner, particularly showing that allowing for so many strategies can actually result in no play being a very successful alternative.

Even though we have focused our analysis on systems in the limit of small mutation rates and without population structure, there is no reason to suspect that the effects we have highlighted will not be salient as well in systems with larger mutation rates [7], or with spatial structure [41]–[46].

Our results call into question the “model-less” approach to mutations in evolutionary dynamics, where given a strategy set, all mutations are available an equally likely. Even in the limit of rare mutations, the mutation structure can make a substantial difference on what gets selected. It is important to observe that all models that follow the methodology studied here, rest on a specific assumption of mutation structure [12]. It is therefore important, not to just consider what the strategy space is, but also if there are natural ways to infer a specific topology or interpretation of the set of strategies in relation to mutations.

## Methods

The evolutionary dynamics is studied based on the Moran process [47]. We consider a finite population of constant size 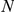. At every time step, one strategy is chosen for reproduction in proportion to its performance in the current population. A copy of this strategy is added to the population after removing a random strategy. With a small probability, the strategy that is copied changes its type to any of the other available strategies. If the strategy selected for reproduction is 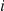, its mutation probability to a strategy 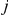 is given by 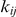. The probability of mutations are summarized in a normalized stochastic matrix 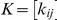, here called mutation kernel. The process results in an ergodic Markov chain [1]. Fitness values are obtained by mapping fitness using an exponential function 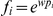, where 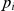 is the payoff of the game being considered; and 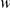 is the intensity of selection.

We asses the effect of selection and mutation by inspecting the average composition of the population in the long run. The stationary distribution can be computed exactly, if mutations are sufficiently small [1], [2]. We have compared the theoretical predictions to Monte Carlo simulations (symbols in the Figures). The stationary distribution is estimated by averaging the result of a sufficient number of runs. Each run is composed of a number of generations, starting in a random population. The average composition of the population is computed during a window at the end of each run.

## Supporting Information

Text S1 Supporting information.
(PDF)
