# Correction: The Structure of Mutations and the Evolution of Cooperation

**DOI:** 10.1371/annotation/7f22fd68-23bf-4c4e-a091-a92090e9314b

**Published:** 2012-07-03

**Authors:** Julián García, Arne Traulsen

The legends for Figures 1 and 2 were incorrectly switched. The legend that appears with Figure 1 should be with Figure 2, and the legend that appears with Figure 2 belongs with Figure 1. The figure images appear in the correct order.

