# Supplementary material for: The Structure of Mutations and the Evolution of Cooperation
Source: PLoS One. 2012 Apr 26;7(4):e35287. doi: 10.1371/journal.pone.0035287 (PMC3338512; doi:10.1371/journal.pone.0035287)
Supplement: Text S1 — Supporting information. (PDF) [file pone.0035287.s001.pdf]

The structure of mutations and the evolution of cooperation Supporting Information Text

Julian Garcia and Arne Traulsen
Research Group for Evolutionary Theory
Max-Planck-Institute for Evolutionary Biology, Plön, Germany
garcia@evolbio.mpg.de, traulsen@evolbio.mpg.de

A Payoff matrix in repeated games with minimal memory strategies

To derive the payoff matrix for all the strategies we compute the normalized payoff, given by

$$\Pi_{AB} = (1-\delta)\sum_{i=0}^{\infty}\delta^i \pi^i_{AB} \tag{1}$$

The complete payoff matrix is then

$$\begin{pmatrix}
R & R & \mathcal{A}(R,S) & \mathcal{A}(R,S) & \mathcal{A}(S,R) & \mathcal{A}(S,R) & S & S \\
R & R & \mathcal{D}(R,S,P,T) & \mathcal{B}(R,S,P) & \mathcal{B}(S,T,R) & \mathcal{C}(S,T) & \mathcal{D}(S,P,T,R) & \mathcal{A}(S,P) \\
\mathcal{A}(R,T) & \mathcal{D}(R,T,P,S) & \mathcal{C}(R,P) & \mathcal{B}(R,P,S) & \mathcal{B}(S,R,T) & \mathcal{D}(S,R,T,P) & S & S \\
\mathcal{A}(R,T) & \mathcal{B}(R,T,P) & \mathcal{B}(R,P,T) & \mathcal{A}(R,P) & \mathcal{A}(S,T) & \mathcal{B}(S,T,P) & \mathcal{B}(S,P,T) & \mathcal{A}(S,P) \\
\mathcal{A}(T,R) & \mathcal{B}(T,S,R) & \mathcal{B}(T,R,S) & \mathcal{A}(T,S) & \mathcal{A}(P,R) & \mathcal{B}(P,S,R) & \mathcal{B}(P,R,S) & \mathcal{A}(P,S) \\
\mathcal{A}(T,R) & \mathcal{C}(T,S) & \mathcal{D}(T,R,S,P) & \mathcal{B}(T,S,P) & \mathcal{B}(P,T,R) & P & \mathcal{D}(P,T,R,S) & P \\
T & \mathcal{D}(T,P,S,R) & T & \mathcal{B}(T,P,S) & \mathcal{B}(P,R,T) & \mathcal{D}(P,S,R,T) & \mathcal{C}(P,R) & \mathcal{A}(P,S) \\
T & \mathcal{A}(T,P) & T & \mathcal{A}(T,P) & \mathcal{A}(P,T) & P & \mathcal{A}(P,T) & P
\end{pmatrix}$$

where have used auxiliary functions that correspond to different cases as follows:

Function $\mathcal{A}$: Distinct behaviour in the first round

Consider the case of *ALLC* playing against *STFT*. Here, in the first round *ALLC* will play co-operate and *STFT* will play defect, getting one-shot payoffs S and T respectively. In subsequent rounds they both cooperate, and each player gets R per interaction. Function A arises whenever the strategy receives payoff x in the first round of the repeated game, and payoff y in all the subsequent stages, it is given by

$$\mathcal{A}(x,y) = x(1-\delta) + y\delta. \tag{2}$$

Note that when $\delta = 0$ only one interaction takes place, thus only x matters. Likewise, when $\delta = 1$ and the game is played forever the first interaction payoff vanishes.

Function $\mathcal{B}$: Distinct behaviour in the first two rounds

In the previous case one round was enough for the strategies to settle on one action. Now consider the case *TFT* playing against *SALLC*. Here the strategies need two rounds before settling. In the first round they will play C and D respectively. In the second round,*TFT* switches to defection and *SALLC* starts cooperating. From the third round on it is all mutual cooperation. So more generally, a strategy gets a payoff x in the first round, a payoff y in the second round and a payoff z from then on. This means that the payoff has the form

$$(1-\delta)\left(x+\delta y+\sum_{i=2}^{\infty}\delta^i z\right). \tag{3}$$

Simplifying, we get an expression for function $\mathcal{B}$

$$\mathcal{B}(x,y,z)=x(1-\delta)+y(1-\delta)\delta+z\delta^2. \tag{4}$$

Function $\mathcal{C}$: Actions cycle with period two

In the previous two cases strategies settle on one action after one or two rounds. We can also have cycling actions, with strategies repeating themselves every few rounds. Consider the case of *TFT* playing against *STFT*. In the first round they cooperate and defect respectively. In the second round they both copy each other's behaviour, defecting and cooperating respectively. The strategies struggle to settle on one action, failing to coordinate the cycle repeats forever. The general form for this payoff is given by

$$(1-\delta)\left(\sum_{i=0}^{\infty}\delta^{2i}x+\sum_{i=0}^{\infty}\delta^{2i+1}y\right). \tag{5}$$

when simplified, we get function $\mathcal{C}$

$$\mathcal{C}(x,y)=\frac{x+y\delta}{1+\delta}. \tag{6}$$

Function $\mathcal{D}$: Actions cycle with period four

Finally, we can get longer cycles. Consider the case of *TFT* playing against TFT^{-1}. Both strategies will start cooperating in the first round, each getting R. In the second round *TFT* copies her opponent's last action, sticking to cooperation while TFT^{-1} reverses her opponent's action switching to defection; *TFT* gets S and TFT^{-1} gets T. In the third interaction *TFT* switches back to defection, and TFT^{-1} reverses the last action of her opponent, playing D; they both get P on mutual defection. In the fourth round *TFT* sticks to defection, and TFT^{-1} switches to cooperation, getting T and S respectively. In round five they both cooperate, starting the cycle again.

Since the one-shot game has four different payoff values this is the longest cycle that a strategy will ever get into. In rounds 0, 4, 8, 12, ... a payoff x is achieved, in rounds 1, 5, 9, 13,... a payoff y is achieved, a payoff z is gotten for rounds 2, 6, 10, 14,... and a payoff w is gotten for rounds 3, 7, 11, 15,... Thus, the payoff has the form

$$(1-\delta)\left(\sum_{i=0}^{\infty}\delta^{4i}x+\sum_{i=0}^{\infty}\delta^{4i+1}y+\sum_{i=0}^{\infty}\delta^{4i+2}z+\sum_{i=0}^{\infty}\delta^{4i+3}w\right). \tag{7}$$

We can simplify this expression to get function $\mathcal{D}$ as

$$\mathcal{D}(x, y, z, w) = \frac{x + y\delta + z\delta^2 + w\delta^3}{1 + \delta + \delta^2 + \delta^3} \tag{8}$$

B Existence and location of stable mixtures

The accuracy of the theoretical prediction depends on non-homogeneous populations being transient [1]. In particular, for the monomorphic transition matrix to be accurate, we require that a new mutation only arises after the population has fixated [2]. A sufficiently small mutation rate guarantees such accuracy if there are no stable internal equilibria between any two strategies in the game. Since mutation is sufficiently small, the dynamics will be confined to the edges of the simplex. This means that we need only inspect all pairs of strategies in the game for possible coexistence.

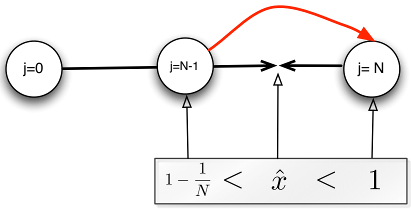

Figure 1: Illustration of condition 9.

It turns out that there are internal mixed equilibria in the the repeated prisoner's dilemma. We can circumvent this difficulty if internal equilibria are close enough to the boundary of a homogeneous state. For any stable mixed equilibria $\hat{x}$ on the edge we require that either

$$1 - \frac{1}{N} < \hat{x} < 1 \tag{9}$$

or

$$0 < \hat{x} < \frac{1}{N} \tag{10}$$

When condition 9 or condition 10 is met, the non-homogeneous states are guaranteed to be transient again, even in the presence of internal equilibria – given a sufficiently small mutation rate (see figure 1 for an illustration). Table 1 summarizes stable mixed equilibria for the game matrix presented in section A.

It is easy to verify that all internal equilibria in Table 1 vanish if $\delta < 1/3$. However, this is a meaningless area of the parameter space, because on average the game is played less than twice, rendering repetition ineffective. We therefore require that for all internal equilibria are sufficiently close to the boundary of a homogeneous population as per equations 9 and 10. For the standard parameters that we analyze $(R = 3.0, S = 0.0, T = 4.0, P = 1.0)$ and $N = 50$, this is guaranteed whenever $\delta \geq \frac{1}{75}\left(24 + \sqrt{2451}\right) \approx 0.980101$. Which means that the choice of $\delta = 0.99$ guarantees the accuracy of the prediction for all conditions in Figure 2 in the main text.

Table 1: Summary of internal equilibria in the repeated prisoner's dilemma with eight strategies.

Strategy A	Strategy B	Internal equilbria $\hat{x}$
ALLC	*STFT*	$\frac{P-R\delta+S\delta-S}{P-2R\delta+R+S\delta-S+T\delta-T}$
TFT^{-1}	*STFT*	$\frac{-P\left(\delta^2+\delta+1\right)+\delta(R+T\delta)+S}{P(\delta+1)^2+R(\delta-1)^2-(\delta^2+1)(S+T)}$
SALLC	*STFT*	$\frac{P\delta+P-S-T\delta}{2P\delta+P+R-(\delta+1)(S+T)}$
NALLD	*STFT*	$\frac{P-R\delta+S(\delta-1)}{P-2R\delta+R+(\delta-1)(S+T)}$
STFT	*STFT*$^{-1}$	$\frac{\delta(P-S\delta)+R((\delta-1)\delta+1)-T}{P(\delta+1)^2+R(\delta-1)^2-(\delta^2+1)(S+T)}$

References

[1] Fudenberg D, Imhof LA (2006) Imitation process with small mutations. J Econ Theory 131: 251-262.

[2] Wu B, Gokhale C, Wang L, Traulsen A (2011) How small are small mutation rates? Journal of Mathematical Biology : 1-25.
